# Alzheimer’s disease diagnosis based on the Hippocampal Unified Multi-Atlas Network (HUMAN) algorithm

**DOI:** 10.1186/s12938-018-0439-y

**Published:** 2018-01-22

**Authors:** Nicola Amoroso, Marianna La Rocca, Roberto Bellotti, Annarita Fanizzi, Alfonso Monaco, Sabina Tangaro, The Alzheimer’s Disease Neuroimaging Initiative, Michael Weiner

**Affiliations:** 1 Dipartimento Interateneo di Fisica “M. Merlin”, Università degli Studi di Bari “A. Moro”, Via Giovanni Amendola 173, 70125 Bari, Italy; 2 Istituto Nazionale di Fisica Nucleare, Sezione di Bari, Via Orabona 4, 70123 Bari, Italy; 3 Istituto Tumori Bari Giovanni Paolo II - IRCCS, Viale Orazio Flacco 65, 70124 Bari, Italy

**Keywords:** Hippocampal Segmentation, Alzheimer’s disease, Neural Networks, Multi-atlas, MCI

## Abstract

**Background:**

Hippocampal atrophy is a supportive feature for the diagnosis of probable Alzheimer’s disease (AD). However, even for an expert neuroradiologist, tracing the hippocampus and measuring its volume is a time consuming and extremely challenging task. Accordingly, the development of reliable fully-automated segmentation algorithms is of paramount importance.

**Materials and methods:**

The present study evaluates (i) the precision and the robustness of the novel Hippocampal Unified Multi-Atlas Network (HUMAN) segmentation algorithm and (ii) its clinical reliability for AD diagnosis. For these purposes, we used a mixed cohort of 456 subjects and their T1 weighted magnetic resonance imaging (MRI) brain scans. The cohort included 145 controls (CTRL), 217 mild cognitive impairment (MCI) subjects and 94 AD patients from Alzheimer’s Disease Neuroimaging Initiative (ADNI). For each subject the baseline, repeat, 12 and 24 month follow-up scans were available.

**Results:**

HUMAN provides hippocampal volumes with a 3% precision; volume measurements effectively reveal AD, with an area under the curve (AUC) AUC_1_ = 0.08 ± 0.02. Segmented volumes can also reveal the subtler effects present in MCI subjects, AUC_2_ = 0.76 ± 0.05. The algorithm is stable and reproducible over time, even for 24 month follow-up scans.

**Conclusions:**

The experimental results demonstrate HUMAN is a precise segmentation algorithm, besides hippocampal volumes, provided by HUMAN, can effectively support the diagnosis of Alzheimer’s disease and become a useful tool for other neuroimaging applications.

## Background

Alzheimer’s disease (AD) is the most common cause of dementia as it accounts for 60–80% of cases [1]. Dementia describes, by definition, memory loss and a variety of other intellectual abilities such as clear thinking. Pathological characteristics of AD are degeneration of specific nerve cells, presence of neuritic plaques and, in some cases, noradrenergic and somatostatinergic systems that innervate the telencephalon [2]. Neuronal loss is not generalized but it privileges specific locations. In fact, one of the best supportive features for AD diagnosis is temporal lobe atrophy and, more importantly, the atrophy of particular sub-cortical structures such as hippocampi [3]. Magnetic resonance imaging (MRI) can be a powerful tool [4, 5], provided that robust fully automated procedures replace current clinical practices, which involves visual inspection [6] and are inherently affected by high inter-rater variability.

Even if the rapid growth of knowledge about the potential pathogenic mechanisms of AD has spawned numerous experimental therapeutic approaches to enter into clinical trials [7, 8], early detection of AD remains far to be achieved as it would require an accurate intervention on subjects affected by mild cognitive impairment, a condition which in some cases is a prodromal AD state, further more difficult to detect. In this case, diagnostic ranges of sensitivity 46–88% and specificity of 37–90% have been reported [9]. These results indicate that many patients not affected at all, or far to be affected, by AD were treated, thus diluting the statistical significance of these trials and the chance to detect a treatment.

Accordingly, more advanced imaging strategies have been recently proposed in search of effective AD markers. Some studies focused on the whole brain [10–14], others preferred the analysis of specific brain regions [15–17]. As a prominent role is played by hippocampus, in this work we investigate the adoption of a specific hippocampal segmentation strategy: the Hippocampal Unified Multi-Atlas Network [18]. HUMAN exploits the accuracy of multi-atlas approaches (representing the state-of-the-art for hippocampal segmentation) and combines it with the robustness of machine learning strategies, thus obtaining an effective and unified segmentation framework. Multi-atlas approaches are based on the use of available labeled scans, in this case with hippocampal manual tracings, to segment unseen scans: labeled examples are usually warped onto the scan to be segmented and segmentation is obtained by label fusion [19]. Multi-atlas approaches have, in fact, some ineradicable drawbacks [20]: registration failures, voxel resampling and thresholding of warped masks are sources of noise affecting the label fusion and the accuracy of segmentations. Classification approaches can improve label fusion [21, 22], this is why recent works have been experimenting a combined strategy [23, 24].

However, the utility of a precise segmentation relies on its clinical application; in order to be useful, segmentations have to reveal the effects of disease. Several works have shown promising results when using hippocampal volumes [25, 26] or subdivisions of the hippocampus [27] for AD diagnosis. Recently, a particular attention has been given to fully automated methods for volume extraction and classification [28]. It is now understood that hippocampal atrophy is a diagnostic marker of AD, even at the MCI stage [4], on the contrary an aspect which is not clear yet is how segmentation precision affects these results. Besides, the application of precise segmentation methods is not limited to AD. Another important field of interest is the monitoring of Multiple Sclerosis lesions.

We present here an evaluation of HUMAN precision with a particular attention to the diagnostic application. To this aim, we explore the information content provided by HUMAN segmented volumes on a mixed cohort from ADNI. The paper is organized as follows: in *Materials and Methods* we provide a synthetic overview of the image processing pipeline and how hippocampal volumes can be used to detect diseased patterns; in *Results* we present our findings; finally, *Discussion* and *Conclusions* summarize our work.

## Methods

### Subjects

Data used in preparation of this article were obtained from ADNI database (adni.loni.usc.edu). The ADNI was launched in 2003 as a public-private partnership, led by Principal Investigator Michael W. Weiner, MD. The primary goal of ADNI has been to test whether serial magnetic resonance imaging, positron emission tomography, other biological markers, and clinical and neuropsychological assessment can be combined to measure the progression of mild cognitive impairment and early Alzheimer’s disease.

For the present study, 456 subjects from ADNI including 145 CTRL, 217 MCI and 94 AD subjects were analyzed. Data consisted of a random sample of 1.5 and 3.0 T1 scans having 4 different time acquisitions: screening, repeat, 12 month and 24 month follow-up scans. The whole training procedure of HUMAN algorithm was performed on an independent training set consisting of a mixed cohort of 100 subjects including 29 CTRL, 34 MCI and 37 AD subjects; the set was selected to be representative of the whole ADNI collection, as it was firstly employed by the EADC-ADNI consortium^1^ to define a novel segmentation protocol of the hippocampus [29]. Demographic information is summarized in the following Table 1.

**Table 1:** Data size, age range and gender are shown for each diagnostic group (CTRL, MCI and AD subjects)

		Size	Age	Gender (M/F)
Training				
CTRL		29	75 ± 7	16/13
MCI		33	74 ± 8	17/16
AD		38	74 ± 8	22/16
Test				
CTRL		145	73 ± 6	78/67
MCI		217	75 ± 9	108/109
AD		94	75 ± 9	51/43

Mean and standard deviation are shown when appropriated. Demographic is reported in different rows for training and test sets

For each subject, screening and repeat scans were acquired with a short time delay (within 4 weeks), thus it was reasonable to assume they were not affected by any significant clinical/morphological change. This assumption is fundamental to evaluate the precision of segmented volumes. Precision of a measurement is by definition the amount of variation that exists in the values of multiple measurements of the same quantity. In brief, as brains should not show any significant morphometric difference, an ideally precise and replicable measure of the hippocampal volume should give identical results. Follow-ups were used instead to investigate the precision of HUMAN segmentations over time, especially to see if the segmentations were able to find known biological relevant aspects.

### Image processing

The HUMAN algorithm performs hippocampal segmentations in three main phases, as detailed in previous work [18]:

1. *Non-linear registration*. The intensity of MRI scans is normalized to lie within the [0,1] range and the eventual bias field is removed before that a non-linear registration (warp) is performed with a data driven template.
2. *Atlas selection*. Pearson’s correlation is measured between the scan to be segmented and the training scans. In this way, optimal atlases are chosen. These atlases are the base of knowledge for subsequent machine learning.
3. *Classification*. From peri-hippocampal regions we extract statistical and textural features; the resulting features are used to train a voxel-based classifier and the final hippocampal segmentation is obtained by label fusion.

**Fig. 1:**
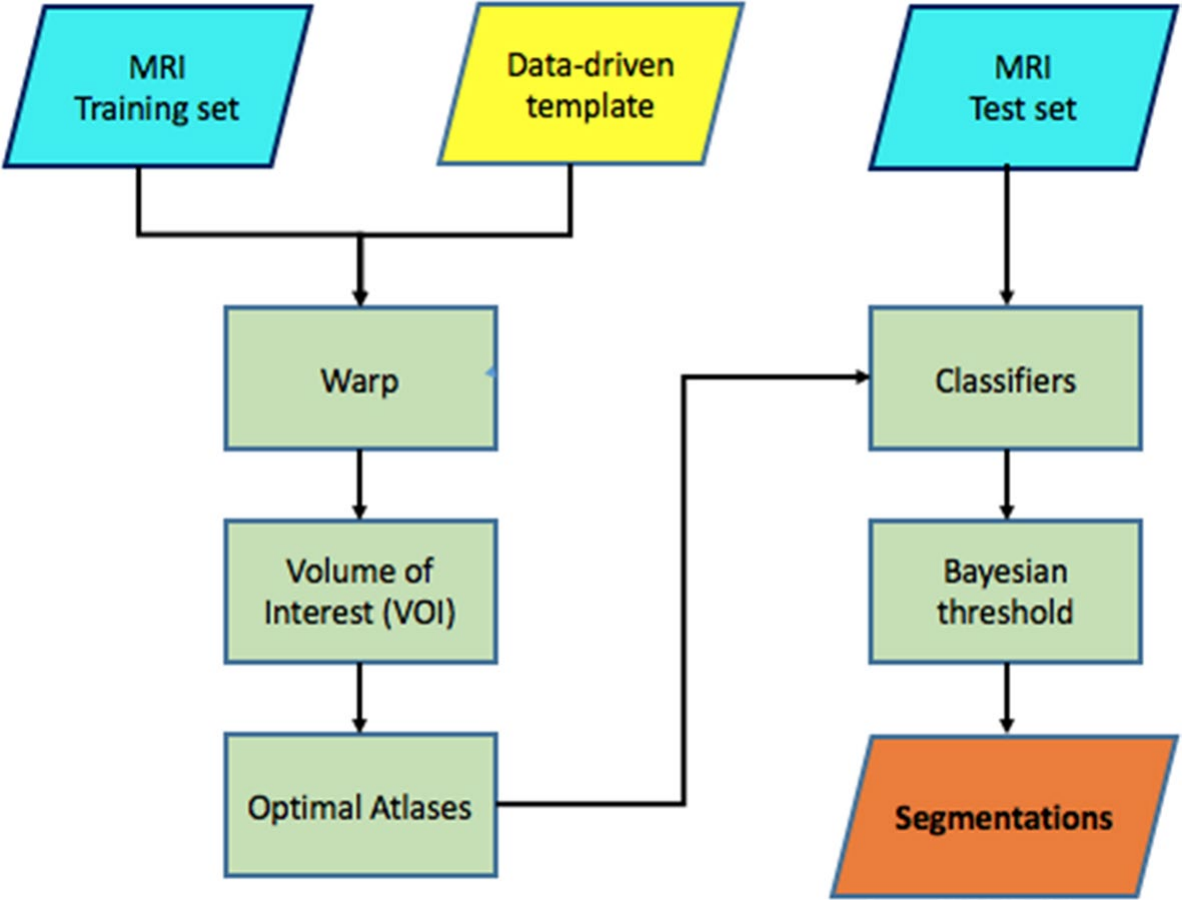
The HUMAN processing pipeline. A data driven template is built from controls, then training scans are warped and peri-hippocampal volumes of interest (VOI) are extracted. VOI is automatically traced on the template, such that hippocampi of warped scans are contained within. The most similar VOIs are used to select optimal atlases; finally, from each scan a neural network is trained to obtain a putative segmentation. The final segmentation is the average of putative segmentations, a Bayesian threshold is used to get a binary response

A synthetic overview is reported in the following flowchart in Fig. 1.

HUMAN algorithm aims at a robust spatial normalization of MRI scans. This is the main prerequisite for a successful segmentation. Firstly, all MRI scans are normalized and the bias field removed with the improved N3 MRI bias field correction algorithm [30], in order to minimize differences in intensity due to the use of different scans or to magnetic field inhomogeneities. To improve registration accuracy we firstly built a data-driven template $\mathcal {T}$ by averaging healthy subjects with the publicly available software Advanced Normalization Tools^2^, which can give accurate average representations from highly variable anatomy. Secondly, we performed a linear registration with FLIRT (FMRIB’s Linear Image Registration Tool) [31]. Lastly, we used again Advanced Normalization Tools to perform non-linear registration [32, 33], the combination of these two registration procedures allowed us to maximize the overlap of different scans improving the hippocampal segmentation.

After registration of scans $\mathcal {S}_i$ to the template $\mathcal {T}$, for multi-atlas segmentation the second crucial step prescribed the selection of optimal atlases. In particular, we extracted from warped MRI scans a common volume of interest (VOI), including the peri-hippocampal region, which was evaluated through a shape analysis algorithm [34] on training scans and automatically traced on the template, such that hippocampi of warped scans were contained within. In brief, the VOI is obtained by assigning to each voxel a probability to belong or not to the hippocampi. We measured in this VOI the pairwise similarity between training and test scans. This step is of paramount importance to reduce the computational burden involved by the procedure and increase the algorithm accuracy. Optimal atlases were chosen by measuring their Pearson’s correlation *r* with the test scan:

1 $$\begin{aligned} r = \frac{N\sum _{j=1}^N x_j y_j - \left(\sum _{j=1}^N x_j\right) \left(\sum _{j=1}^N y_j\right)}{\sqrt{\left[N\sum _{j=1}^N x_j^2 - \left(\sum _{j=1}^Nx_j\right)^2\right]\left[N\sum _{j=1}^N y_j^2 - \left(\sum _{j=1}^N y_j\right)^2\right]}} \end{aligned}$$

the sum is extended to all *N* voxels in the peri-hippocampal VOI; $x_j$ represents the intensity of the *j*-th voxel of a training scan, $y_j$ is the intensity of the corresponding *j*-th voxel in the test scan. The most similar scans were the ten scans with higher correlations.

From each voxel within the VOI we extracted statistical and textural features. Statistical features included the average, the standard deviation and other central moments computed on square boxes with varying size (from $3 \times 3 \times 3$ to $9 \times 9 \times 9$ voxels) and centered on the voxel of interest. We also computed textural features such as the Haralick and Haar-like features [35–37].

We fed a neural network model for each VOI. The optimal configuration consisted of neural networks trained with the backpropagation algorithm with one hidden layer containing ten neurons and standard sigmoid activation functions. These models learned to distinguish hippocampal voxels from background according to the computed features. To segment a test scan we finally used a weighted average; using only models corresponding to optimal atlases and their measured correlations as weights, we assigned to each voxel within the test scan VOI a classification score.

The test segmentation was finally obtained by a threshold determined with a Bayesian approach. Firstly, we determined on training the *a priori* probability for a voxel to belong to the hippocampus *P*(*H*). Secondly, we estimated with repeated 5-fold cross-validations the training classification sensitivity *S* and specificity *s*. Finally, we obtained the desired threshold as the *a posteriori* probability *t*:

2 $$\begin{aligned} t = \frac{S \cdot P(H)}{S \cdot P(H) + (1 - s) \cdot (1 - P(H))} \end{aligned}$$

Each voxel with a classification score exceeding this threshold was assigned to the hippocampus. Further details about algorithmic and computational aspects concerning HUMAN are presented and discussed in our previous study [18].

Following this procedure we segmented 1824 scans, for both left and right hippocampi, 456 scans for each time point. These segmentations provided the hippocampal volumes which were used for the two-class discrimination problems: CTRL–AD and CTRL–MCI.

### Alzheimer’s disease classification

ADNI database does not include ground truth segmentations of hippocampi, so that it is not possible to perform a direct evaluation of segmentation accuracy. Nevertheless, it is possible to obtain an indirect measure, at least from a clinical perspective. Segmentation algorithms are usually evaluated in terms of error metrics, such as Dice index, Hausdorff distance, Recall. These metrics are useful to measure the agreement of segmentations with manual tracings provided by human experts. However, these metrics do not measure whether and how much these segmentations are associated to the diagnosis, an aspect which is fundamental for clinical applications.

**Fig. 2:**
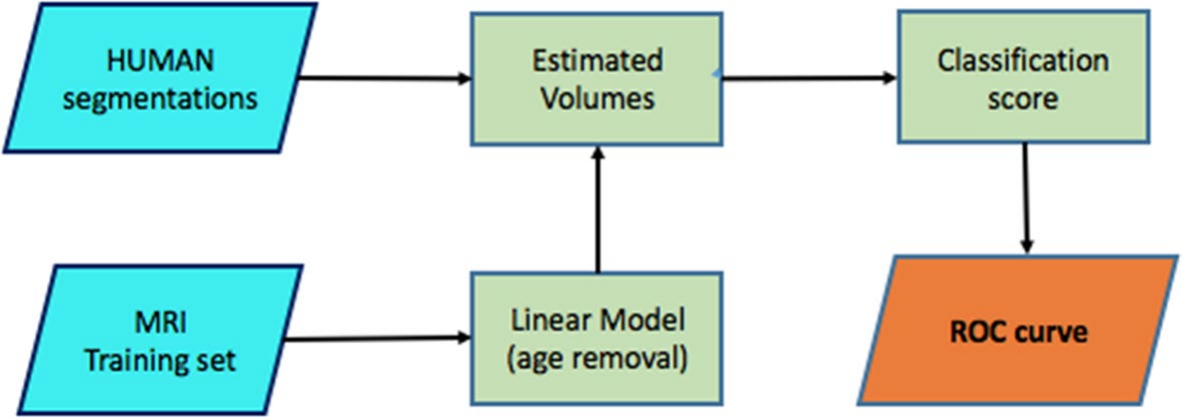
The classification flowchart with HUMAN segmentations. Age effect is removed from measured volumes, then these measurements are used as a classification score: a volume threshold is established, the threshold assigns the subjects to two distinct classes (CTRL/AD and CTRL/MCI). Finally, a receiver operating characteristic (ROC) curve is computed, determining the number of true positives and false positives

To evaluate the informative content of HUMAN segmentations and their predictive power in order to detect AD, we used hippocampal volumes as diagnostic indexes. The procedure is shown in Fig. 2.

It is known that hippocampal volumes are a supportive feature for probable AD diagnosis, thus a well performing segmentation algorithm must return a volume distribution which significantly separates the CTRL, MCI and AD cohorts. Besides, to evaluate how good is the separation, volumes were used to build a simple receiver operating characteristic (ROC) curve, for both CTRL–AD and CTRL–MCI classification tasks. With a varying volume threshold, we measured the true positive rate (AD or MCI subjects correctly classified with the given) against the false positive rate (CTRL subjects incorrectly classified at the same threshold); thus we built the ROC curve.

To help classification, we removed the normal aging effect from volumes with a linear regression model. As reported by several studies [38, 39] normal aging has an atrophy effect which for hippocampi has an estimated value of about 30 mm^3^ per year. Accordingly, we built a linear model to describe the estimated hippocampal volumes $\hat{V}$ as a function of the subject age and using only the training CTRL cohort:

3 $$\begin{aligned} \hat{V} = V_0 + k(t-t_0) \end{aligned}$$

We observed an angular coefficient $k = -29.9$ mm^3^ per year with a 95% confidence interval [29.2, 30.5] mm^3^ per year and an intercept value $V_0 = 3173.0$ mm^3^. These values resulted in an accurate fit with $R^2 = 0.89$. The age effect was then removed from each measured volume *V*, thus obtaining an *effective* volume *V*_eff_ for each generic age *t*:

4 $$\begin{aligned} V_{eff} = \hat{V} - V \end{aligned}$$

The reference time (measured in years) $t_0$ was set to be the minimum age of the whole cohort. In this way we removed atrophy effects due to normal aging.

Finally, we used these volumes as diagnostic scores and computed the related receiver operating characteristic (ROC) curves for the two binary classification tasks CTRL–AD and CTRL–MCI. We measured the informative content in terms of AUC. We investigated in this way the robustness of the segmentation results and the effectiveness of hippocampal volumes as discriminant features of AD.

## Results

### Evaluation of HUMAN precision

**Fig. 3:**
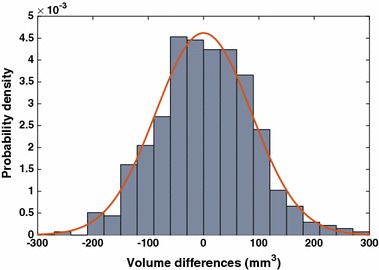
The distribution of differences between screening and repeat segmented volumes. The distribution of residuals shows a normal behavior consistent with a null mean ($1.4 \pm 84.3$ mm^3^). In red the Gaussian distribution derived from the data

A valid measure system should be both accurate and precise as a not precise measure would be affected by a large uncertainty, although remaining on average accurate. From a clinical point of view an accurate but not precise segmentation algorithm is unreliable. To measure HUMAN precision (even without available repeated acquisitions), we considered screening and repeat scans of the same subject indistinguishable, then we investigated the distribution of volume residuals $V_\text{{screening}} - V_\text{{repeat}}$. Results are shown in the following Fig. 3.

As no morphometric change can occur between the screening and the repeat MRI acquisitions, all volumetric differences observed must descend from the algorithm intrinsic uncertainty. No systematic bias was observed; the mean value of residuals was $1.4 \pm 84.3$ mm^3^, which was consistent with a null average and small if compared to the average hippocampal volume (considering that training hippocampi had a mean volume of 2650.2 mm^3^). It is worthwhile to note that the volume differences were calculated from different subjects, nonetheless it is reasonable to assume that the algorithm precision on a large sample should remain constant for all subjects. Accordingly, we considered the standard deviation of residuals $\sigma = 84.3$ mm^3^ an indirect measure of the algorithm precision. Compared to the mean hippocampal volume of 2650 mm^3^, the measured precision represented a $3\%$ of the whole hippocampus.

The narrow distribution of volume residuals is not sufficient to prove the consistency of different segmentations, as for example it gives no clues about the homoscedastic or heteroscedastic behavior of the methodology. This is important especially to determine whether the algorithm precision varies with the volume to be segmented. In this sense, further information is provided by a correlation analysis. In fact, we measured the Pearson’s correlation between baseline and repeat segmented volumes, then we performed the same pairwise correlation analysis for all available time points. Also, we investigated the volume distribution at each time point.

**Fig. 4:**
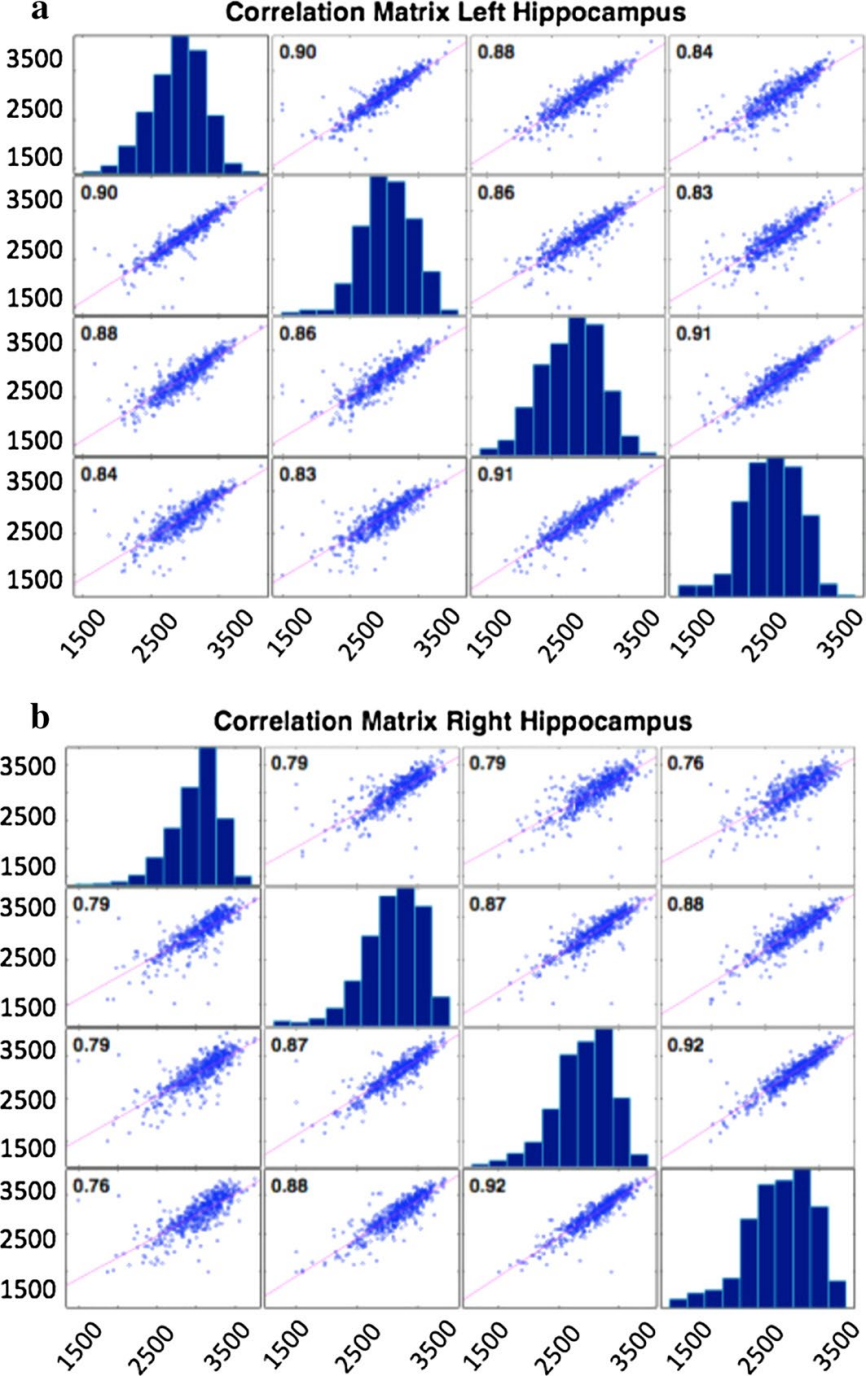
Correlation plots. The figure shows the correlation plot for left (**a**) and right (**b**) hippocampal volumes: volumes at each time point are plotted against other time point volumes, the main diagonal represents the volume distributions. Correlations are computed for all time points considering both screening and repeat scans. The analysis shows high correlations, a proof of the segmentation algorithm consistency

Baseline and repeat scans showed a high correlation for both left $r = 0.90$ and right $r = 0.79$ hippocampi. Interestingly, higher correlations were found considering follow-ups. In particular, as shown in Fig. 4, the highest values were found for correlations between 12 and 24 month follow-ups; we found $r = 0.91$ and $r = 0.92$ respectively for left and right cases.

A strong correlation, demonstrates the good agreement between the measurements. In all examined cases, except for baseline right hippocampi, correlations remained very strong exceeding the commonly adopted, even if rather arbitrary, 0.80 threshold [40]. Moreover, as variance remained almost constant through the whole volume range, the measure is homoscedastic.

### HUMAN segmentations for AD diagnosis

Measuring the precision was necessary to evaluate the clinical utility of the proposed segmentation tool. To evaluate the diagnostic content for a single subject prediction, we built a linear model representing the volume distribution of the CTRL cohort as a function of time and the relative $95\%$ confidence interval. Then we compared the AD volumes using precision as the inherent uncertainty with this model.

**Fig. 5:**
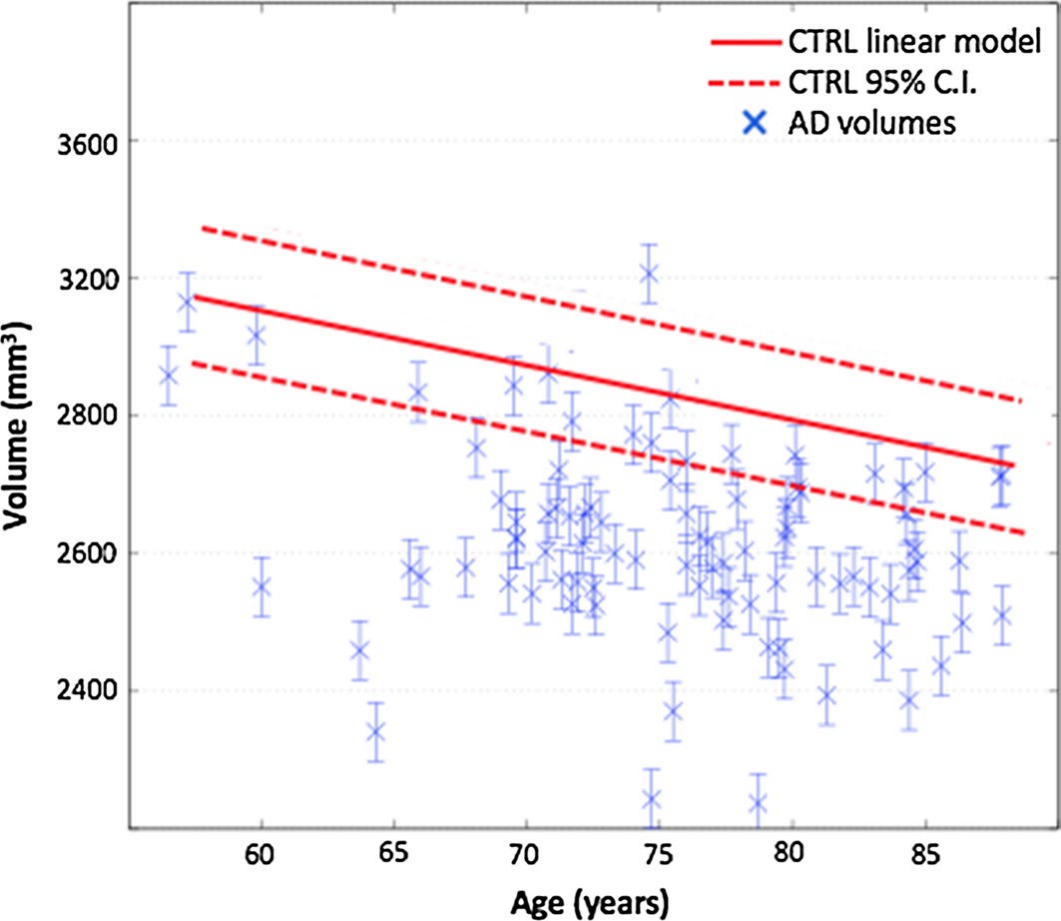
Hippocampal volume variation over time. The linear model describing how hippocampal volumes from healthy subjects vary over time. HUMAN volumes of AD patients are also represented to qualitatively show the informative content of the measurements. In fact, AD subjects show a consistent reduction of the hippocampal volume compared to CTRL expected volumes

As shown in Fig. 5, the hippocampal volumes of AD subjects showed a consistent reduction compared to the CTRL cohort.

**Fig. 6:**
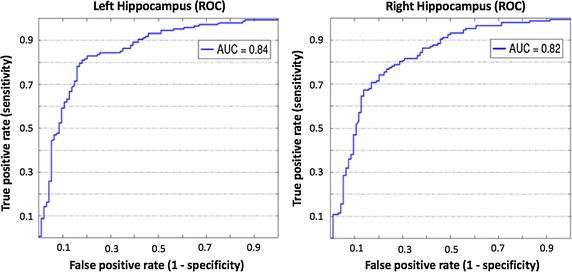
The ROC curves. The ROC curve obtained using the baseline volumes of CTRL and AD subjects as a classification score. The performance is measured in terms of AUC. Raw hippocampal volumes provide robust discrimination for both left and right hippocampi, respectively with AUC$_\text{left} = 0.84 \pm 0.02$ and AUC$_\text{right} = 0.82 \pm 0.02$

Also, we performed a quantitative evaluation of the predictive power of HUMAN segmentations. Using normalized hippocampal volumes as classification scores we could suitably determine the informative power contained in this feature. As a performance measure we used the AUC and bootstrapped the volumes 500 times to get an estimation of the standard error. The following Fig. 6 shows the ROC curves for mixed cohorts of CTRL and AD subjects, both for left and right hippocampi.

Left hippocampi allowed a slightly more accurate discrimination capability with an AUC$_\text{left} = 0.84 \pm 0.02$ (AUC$_\text{right} = 0.82 \pm 0.02$). The standard error of the AUC was calculated with the Hanley-McNeil formula [41]. These results were obtained by considering the raw hippocampal volumes without removing the age confounding effect. In fact, using the proposed linear age detrending a significant improvement of performance was observed. A summary of these improved classification performances for screening, repeat, 12 month and 24 month follow-ups is reported in the subsequent Table 2.

**Table 2:** Table reports the classification performance averaged for left and right hippocampal volumes for two distinct classification tasks: CTRL–AD and CTRL–MCI

Acquisition	AUC CTRL/AD	AUC CTRL/MCI
Screening	0.88 ± 0.02	0.76 ± 0.05
Repeat	0.86 ± 0.03	0.75 ± 0.04
12 month follow-up	0.90 ± 0.02	0.80 ± 0.03
24 month follow-up	0.90 ± 0.01	0.80 ± 0.03

**Fig. 7:**
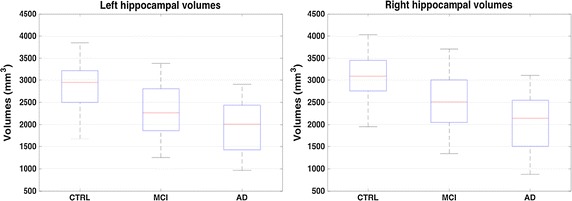
Boxplot of left and right hippocampal volumes. The boxplot of left and right hippocampal volumes divided by clinical status CTRL–MCI–AD. A Kruskal-Wallis test confirmed that the three groups were significantly different *p* < 0.01

In Table 2 the classification performance for the task CTRL–MCI is also reported. In this latter case hippocampal volumes still have a high discriminant power although significantly lower that for CTRL–AD. This is a direct effect of the progressive atrophy affecting the brain, as shown in Fig. 7. A statistical analysis was performed with a non parametric Kruskal-Wallis test; we found a significant difference *p* < 0.01 between hippocampal volumes of CTRL, MCI and AD populations. This result was confirmed for both left and right hippocampi.

As expected, the right volumes were slightly greater than the left ones, a direct effect of the well known AD left-privileging asymmetry. Analogous findings were obtained with screening and repeat scans. Again, the same statistical test confirmed a significant difference for 12 and 24 month follow-ups. To evaluate the informative content provided by hippocampal volumes, we measured the classification accuracy obtainable by determining the class of each subject (CTRL, MCI or AD) using these volumes as discriminative features of a Naive Bayes classifier, see Table 3.

**Table 3:** The three-class (CTRL, MCI and AD subjects) classification performance

Performance	$V_\text{left} + V_\text{right}$	$V_\text{left}$	$V_\text{right}$
Sensitivity	$0.53 \pm 0.08$	$0.50 \pm 0.06$	$0.52 \pm 0.06$
Specificity	$0.76 \pm 0.04$	$0.75 \pm 0.04$	$0.74 \pm 0.04$
Accuracy	$0.50 \pm 0.01$	$0.49 \pm 0.01$	$0.49 \pm 0.01$

Performance was evaluated with a ten-fold cross validation procedure; we performed 100 cross-validation rounds using the sum of left and right hippocampal volumes to feed the classifier and compute the classification accuracy. Then, we performed the same test using only the left hippocampal volume; finally, the right hippocampus was used.

The classification accuracy for the CTRL, MCI and AD classes is simply the number of correct classified examples over the whole sample; the best results were obtained using both hippocampal volumes with a $0.50 \pm 0.01$ accuracy. Besides, to ease the interpretability of results, we considered sensitivity and specificity looking at AD patients as the true positive and MCI and CTRL subjects as true negatives. Accordingly, results showed the hippocampal volumes tend to be a more specific (specificity $\sim 0.75 \pm 0.04$) than a sensitive (sensitivity $0.52 \pm 0.07$) feature.

## Discussion

Our previous work [18] presented HUMAN segmentation methodology and evaluated its reliability in terms of segmentation accuracy. We demonstrated that HUMAN was able to reach an accurate Dice index performance on a manually labeled set of ADNI scans ($0.929 \pm 0.003$) and a comparable result on an independent set whose labels had been provided following a different segmentation protocol ($0.869 \pm 0.002$). In this work, we investigated its diagnostic application thus examining how hippocampal volumes segmented by HUMAN could be related to the diagnosis of ADNI subjects. We demonstrated that using HUMAN volumes it was possible to obtain an accurate classification rate of ADNI subjects, an indirect proof of HUMAN reliability. First of all, we presented a precision analysis, which was fundamental to evaluate the clinical information carried out by HUMAN segmentations. Precision should not be confused with accuracy, even if closely related. Under the same conditions and with sufficient statistics, repeated measurements should be normally distributed around their average; then, accuracy and precision can be measured: accuracy is the difference between the measurement average and a reference value, precision is the spread of the measurement distribution, *i. e.* its standard deviation (for Gaussian distribution). However, due to the particular nature of segmentation problems, the latter tends to be frequently disregarded, especially for image processing oriented works. This work proposes a method to measure the segmentation precision.

To achieve this goal, we hypothesized that screening and repeat scans, being acquired with a short time difference, could ideally considered two independent measurements of an indistinguishable quantity. Therefore, no difference between the segmentation volume of screening and repeat scans should be observed except for statistical uncertainty. In this sense, the observed uncertainty value for residual distribution ($3\%$) demonstrates HUMAN to be a valid segmentation algorithm, accurate and precise.

Moreover, considering the different available time points, a correlation study allowed us to estimate how much the methodology was stable from a longitudinal perspective. A robust segmentation algorithm must return highly correlated hippocampal volumes, even if, after 12 or 24 months, subjects are affected by physiological or pathological atrophy. HUMAN resulted in fact longitudinally robust. All time points, except one, showed a high Pearson’s correlation ($r >0.80$). The correlation observed for left hippocampi resulted significantly higher than for right ones. A possible interpretation of this effect is that left hippocampal volumes are more severely affected by atrophy than right ones; as a consequence, left hippocampal volumes tend to be homogeneous as natural variability is dominated by atrophy. On the contrary, for right hippocampi, less affected by a severe atrophy, natural variability yields a more heterogeneous behavior resulting in a correlation drop particularly remarkable for screening and repeat scans. This interpretation is consistent with correlation results of other time points. Higher correlations were found between 12 and 24 month follow-ups with equivalent values for left and right hippocampi. When atrophy dominates the aging effect, natural heterogeneity is eliminated, thus resulting in an increased segmentation agreement, what is not observed at the baseline when natural variability remains a not negligible confounding factor.

Finally, the presented results demonstrate the usefulness of HUMAN segmentations for diagnostic purposes. In fact, basing only on hippocampal volumes, classification AUC measurements achieve sound results. As expected, the informative content of left hippocampi is slightly but significantly higher than right ones. The result is confirmed for all time points and for both classification tasks: CTRL–AD and CTRL–MCI, the latter with a lower performance. MCI has of course intrinsically subtler differences from CTRL than AD, however another reason behind this performance drop is that MCI can include a wide range of heterogeneous conditions not necessarily leading to AD.

The results of this work demonstrated on one hand the effectiveness of HUMAN hippocampal volume measurements for AD detection, reaching classification performances usually obtainable only with refined machine learning strategies [14] or including wider knowledge domains [13]. These performances compare well with other results reported in literature, see for example a recent international contest launched on the Kaggle platform^3^ reporting classification accuracy about 0.35 for a four class classification (CTRL, AD, MCI and MCI converter). In fact, it should be considered that, among image-based markers, hippocampal volume could play a pivotal role in discriminating population at risk [42]. Classification accuracies reported in literature compare well with the presented results; for example, [43] found an $82\%$ correct classification rate for AD and CTRL subjects and a $64\%$ accuracy when considering CTRL and MCI subjects, which will convert to AD. Analogously, in [44] the correct classification rate for AD and CTRL subjects was about $80\%$ while the accuracy $65\%$ was obtained with MCI subjects. More recently, [45] showed that, integrating longitudinal information (i.e. observing the hippocampal atrophy rate over time) with the baseline segmentation volume, more accurate classification results could be achieved: the discrimination ability gave an area under the curve 0.93 for CTRL–AD classification and 0.88 for CTRL–MCI. It is worth mentioning that in this case, the classification results obtained with HUMAN segmentations show minor accuracies, but using only the information obtainable at the baseline and not including longitudinal information arising from follow-up scans.

It is worth noting that the goal of this work was aimed at measuring the informative power of the hippocampal volumes segmented with the proposed methodology more than offering a comprehensive computer aided detection system for AD; a goal that would surely benefit from the use of additional information as cognitive scores, other atrophy measurements or refined classification strategies. Finally, the precision reported will hopefully stimulate the application of the proposed methodology to other neuroimaging challenging tasks, where the role of precision is of paramount importance; an important application, we intend to investigate, is the automated detection of Multiple Sclerosis lesions and the monitoring of their longitudinal evolution.

## Conclusions

In this work we examine and assess in detail the reliability of the HUMAN method from a clinical perspective. The results demonstrated that the segmentation algorithm is stable and precise ($3\%$), accordingly HUMAN is a reliable tool for hippocampal segmentation and could be suitably adopted to large trials or segmentation protocol evaluation studies.

The use of segmented volumes as classification scores for CTRL–AD discrimination allowed us to measure the informative content associated to this feature, for both left and right hippocampi. Removing the age confounding effect, segmented volumes revealed AD with an AUC$_{1} = 0.88 \pm 0.02$. Besides, also for the CTRL–MCI classification task a sound performance was achieved, AUC$_{2} = 0.76 \pm 0.05$. For future work, it could be interesting to investigate a cohort not including generic MCI subjects, but specifically those converting to AD. This could be in fact a decisive information for early detection of Alzheimer’s disease.

## Footnotes

1 https://www.hippocampal-protocol.net.

2 http://picsl.upenn.edu/software/ants/.

3 https://www.kaggle.com/c/mci-prediction/leaderboard.
